# Prevalence and Clinical Characteristics of Patients With Pseudogout and Concomitant Monosodium Urate Crystals

**DOI:** 10.7759/cureus.115075

**Published:** 2026-08-24

**Authors:** Yuka Masuyama, Sumire Suzuki, Shigeru Onishi, Hiromi Akiba, Richard H Kaszynski, Takuma Sasaki, Yukino Itami, Mikio Nakajima

**Affiliations:** 1 Emergency and Critical Care Center, Tokyo Metropolitan Hiroo Hospital, Tokyo, JPN; 2 Department of Clinical Laboratory, Tokyo Metropolitan Hiroo Hospital, Tokyo, JPN

**Keywords:** cppd, gout, monosodium urate, polarized light microscopy, pseudogout, uric acid

## Abstract

Objective: Pseudogout and gout are crystal-induced arthritides caused by calcium pyrophosphate dihydrate (CPPD) and monosodium urate (MSU) crystals. Reported coexistence rates are generally low (<6%), but prior studies mainly evaluated CPPD deposition in gout cohorts. The prevalence and clinical relevance of concomitant MSU crystals in pseudogout remain unclear. We aimed to determine the coexistence rate of CPPD and MSU crystals in patients with pseudogout and compare clinical characteristics and outcomes by coexistence status.

Methods: This single-center retrospective cohort study was conducted at a tertiary emergency medical center in Tokyo. Consecutive patients diagnosed with pseudogout between April 2018 and November 2025 who underwent synovial fluid aspiration were included. CPPD crystals were identified by polarized light microscopy with simultaneous MSU assessment. Patients were classified into pseudogout-only and gout-superimposed groups. Clinical features, treatments, and time to improvement were compared between the two groups.

Results: CPPD crystals were identified in 107 patients: median age, 86 years; interquartile range, 80-90 years; female sex, 79 (74%). Most patients had monoarticular disease, and the knee was most frequently involved in 92 patients (86%). MSU crystals were detected in 17 patients (15.9%), while 90 (84.1%) had pseudogout alone. Except for higher serum uric acid levels in the gout-superimposed group, no significant differences were found between groups in clinical characteristics, treatment, or outcomes. In coexistence cases, CPPD crystals predominated on polarized light microscopy.

Conclusions: MSU crystal coexistence was observed in 15.9% of patients with crystal-proven pseudogout, exceeding previously reported rates. Systematic assessment for MSU crystals during synovial fluid microscopy may facilitate recognition of concomitant gout in patients with pseudogout; however, prospective studies are needed to determine whether such identification changes clinical management or improves patient outcomes.

## Introduction

Crystal-induced arthritis encompasses two major entities: gout and pseudogout. Pseudogout is caused by the deposition of calcium pyrophosphate dihydrate (CPPD) crystals within the joint. Pseudogout predominantly affects elderly women with degenerative joint changes and is frequently associated with osteoarthritis, prior joint trauma, or previous surgery, and commonly involves large proximal joints such as the knee and upper limbs [1]. Metabolic abnormalities such as hyperparathyroidism and hypomagnesemia, as well as hereditary factors, have been implicated in its pathogenesis [2]. CPPD disease is also recognized as a relatively common cause of fever of unknown origin in hospitalized patients, underscoring the importance of careful joint assessment in cases of acute, unexplained fever.

In contrast, gout results from the accumulation of monosodium urate (MSU) crystals and occurs in middle-aged individuals, with the male-to-female ratio consistently remaining around 3:1 [3]. A recent Japanese study reported that the mean age of patients with gout was 42 years [4]. In a large Chinese cohort study (n=10,658), the mean age at onset declined from 42 to 39 years over a two-decade period, suggesting a gradual shift toward younger onset [5]. The development of gout is influenced by hyperuricemia, alcohol intake, and familial predisposition [6]. Gout typically involves the toes, fingers, and particularly the first metatarsophalangeal joint. While both conditions share a mechanism of crystal-induced inflammation, the literature addressing their coexistence remains limited, consisting mainly of small studies in gout populations in which CPPD deposition was identified incidentally [7,8].

Previous studies on the coexistence of MSU and CPPD crystals have almost exclusively focused on patients with gout. Previous studies, as well as isolated case reports, have reported coexistence rates generally <6%. To date, no large-scale studies have evaluated the presence of MSU crystals in patients initially diagnosed with pseudogout. Consequently, the frequency and clinical characteristics of crystal coexistence among patients with pseudogout remain poorly examined [7,9-11].

Therefore, the primary aim of the present study was to determine the prevalence of concomitant MSU crystals among patients with crystal-proven pseudogout. We also compared clinical characteristics and outcomes between patients with pseudogout alone and those with concomitant MSU crystals.

## Materials and methods

Study design and ethics approval

The present study was a single-center retrospective cohort study conducted at Tokyo Metropolitan Hiroo Hospital, which is located in central Tokyo and is a tertiary-care emergency medical center. The facility is a general hospital with departments of internal medicine, orthopedics, and emergency medicine. This study was approved by the institutional review board at Metropolitan Hiroo Hospital (approval number, JIN, 2025-b-1). Due to the anonymous nature of the data, the requirement for patient informed consent was waived.

Patients

**Figure 1:**
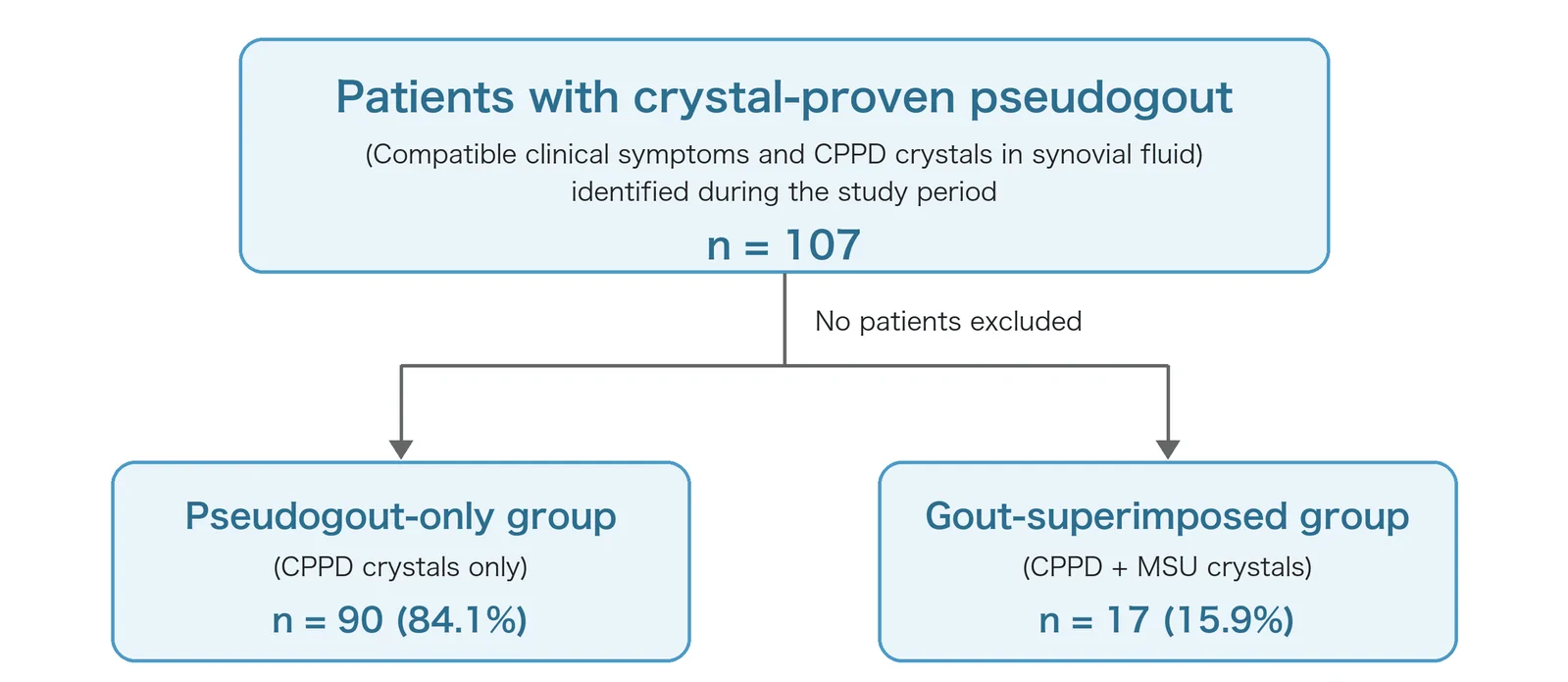
Flow diagram of patients with crystal-proven pseudogout included in the analysis CPPD, calcium pyrophosphate dihydrate; MSU, monosodium urate

We included consecutive patients with compatible clinical symptoms and CPPD crystals identified in synovial fluid between April 2018 and November 2025 at our institution. The diagnosis of pseudogout was based on the identification of CPPD crystals in joint aspiration samples under a polarized light microscope. All joint fluid examinations were performed based on clinical necessity; no additional tests or procedures were conducted for the study. Data were retrieved and compiled from the medical record database. When multiple aspirations were performed in the same patient, the first episode in which crystals were detected was used for analysis. When multiple joints were involved, the number of affected joints was recorded. Patients included were outpatients as well as inpatients. Synovial fluid aspirates were centrifuged, and the sediment was placed directly on a glass slide and examined without fixation or staining by polarized light microscopy using a dedicated gout analyzer (U-GAN, Olympus Corp, Tokyo, Japan) for CPPD and MSU crystals. Eligibility required compatible clinical symptoms and identification of CPPD crystals in synovial fluid; radiographic chondrocalcinosis was not required. The American College of Rheumatology/European League Against Rheumatism gout classification criteria were not applied. No exclusion criteria were specified; thus, all eligible patients were included. The study flow is presented in Figure 1.

**Figure 2:**
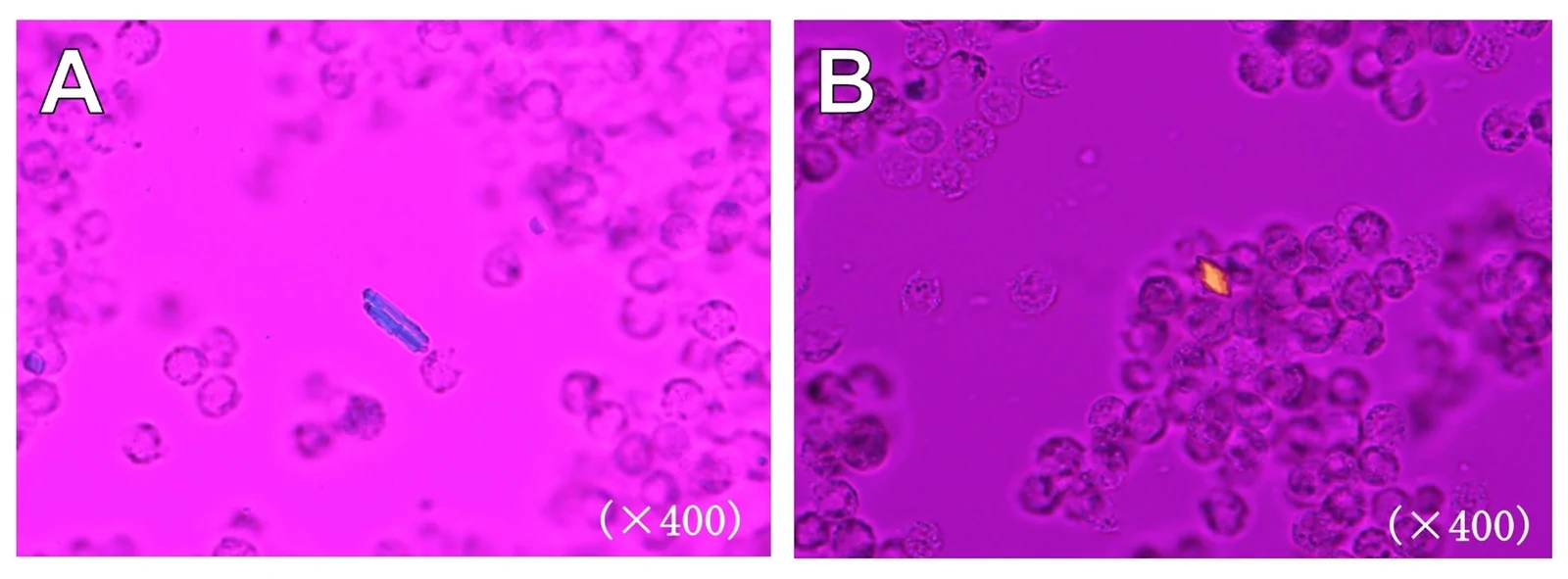
Typical findings of polarized light microscopy in the gout-superimposed group A: Calcium pyrophosphate dihydrate crystals observed under polarized light microscopy. B: Monosodium urate crystals observed under polarized light microscopy.

Patients were divided into two groups: those with clinically diagnosed pseudogout (pseudogout-only group) and those diagnosed with concomitant pseudogout and gout (gout-superimposed group). In the present study, group assignment was determined according to the presence of crystal deposition. The diagnosis of gout was based on the identification of MSU crystals in joint aspiration samples under polarized light microscopy. Crystal identification by polarized light microscopy was performed by clinical laboratory technologists at our institution. CPPD crystals are blunt rods and exhibit weak positive birefringence, whereas MSU crystals are needle-shaped and exhibit strong negative birefringence, allowing differentiation. Typical findings of polarized light microscopy are shown in Figure 2. Even in cases where both CPPD and MSU crystals were present, concurrent visualization within a single microscopic field was uncommon. Coexistence was frequently detected only through careful examination of multiple fields.

Characteristics and outcomes

We described clinical information, including age, sex, medical history, site of onset, maximum body temperature, blood test results at the time of puncture, and X-ray findings obtained from medical records. Outcomes included treatments, C-reactive protein (CRP) levels on days 3, 5, and 7 after symptom onset, and duration of symptoms (time from symptom onset to clinical improvement). These variables were compared between the pseudogout-only and gout-superimposed groups.

Statistical analysis

Continuous variables were presented as medians with interquartile ranges (IQRs), and categorical variables were reported as counts and percentages. The prevalence estimate was reported with a Clopper-Pearson exact 95% confidence interval (CI). Comparisons of continuous variables between groups were performed using the Mann-Whitney U test, whereas categorical variables were analyzed using Fisher’s exact test. A two-sided p-value of less than 0.05 was considered statistically significant. Cases with missing values were not excluded, and summary statistics were calculated based on the available data. CRP values were assessed on days 3, 5, and 7 after symptom onset. When values on these specific days were unavailable, the missing CRP values were supplemented using the nearest available measurements, such as the value on the closest day or the mean of the immediately preceding and following values. All analyses were conducted using StataNow/BE 19.5 (StataCorp, College Station, TX, USA).

## Results

A total of 107 patients were found to have CPPD crystals in synovial fluid. The prevalence of concomitant MSU crystals among these patients was 15.9% (17 of 107 patients; 95% CI, 9.5% to 24.2%). The median age of the overall cohort was 86 years (IQR, 80-90 years), and the population consisted of 28 men and 79 women (male-to-female ratio, 1:2.8). The median number of affected joints was one, with most patients presenting with monoarticular arthritis. The knee was the most frequently involved joint, accounting for 92 patients (86.0%). Other affected sites included the left ankle (seven patients), right ankle (three patients), right shoulder (three patients), right wrist (two patients), and one patient each with involvement of the bilateral elbows, right middle finger distal interphalangeal joint, and right toe distal interphalangeal joint. Among the 107 patients, 90 (84.1%) were classified into the pseudogout-only group, whereas 17 (15.9%) were classified into the gout-superimposed group. There were no statistically significant differences between the two groups with respect to age, sex, comorbidities, site of involvement, body temperature, or radiographic features. Among laboratory findings, serum uric acid levels were significantly higher in the gout-superimposed group than in the pseudogout-only group (P=0.048), whereas other laboratory values did not differ significantly. In the gout-superimposed group, 11 patients (64.7%) were inpatients and six patients (35.3%) were outpatients. The median age was 87 years (IQR, 83-91 years), with five men and 12 women (male-to-female ratio, 1:2.4) (Table 1). The knee remained the most affected joint in this group, accounting for 13 patients (76.5%). None of the patients had a prior history of gout, and all cases represented the first clinically recognized episode of gout. Although seven patients in the pseudogout-only group had a history of gout or hyperuricemia, none had received urate-lowering therapy at the time of presentation. In the gout-superimposed group, polarized light microscopy showed that CPPD crystals appeared more abundant than MSU crystals in most synovial fluid specimens, with MSU crystals observed sparsely among predominant CPPD deposition.

**Table 1:** Patient characteristics Data are shown as numbers (%) and median (IQR) unless otherwise specified. P-values were calculated using the Mann-Whitney U test for continuous variables and Fisher's exact test for categorical variables. A two-sided P-value of less than 0.05 was considered statistically significant. The test statistic shown is the z-value from the Mann-Whitney U test; Fisher's exact test does not yield a test statistic (-). IQR, interquartile range; CRP, C-reactive protein

Variables	Total (n=107)	Pseudogout-only group (n=90)	Gout-superimposed group (n=17)	P-value	Test statistic
Age, years	86 (80-90)	85.5 (80-90)	87.0 (83-91)	0.32	-0.99
Sex, male	28 (26.2%)	23 (25.6%)	5 (29.4%)	0.77	-
On admission	65 (60.7%)	54 (60.0%)	11 (64.7%)	0.79	-
Comorbidities					
Diabetes mellitus	31 (29.0%)	24 (26.7%)	7 (41.2%)	0.25	-
Hypertension	75 (70.1%)	64 (71.1%)	11 (64.7%)	0.58	-
Atrial fibrillation	15 (14.0%)	12 (13.3%)	3 (17.6%)	0.7	-
Cancer history	18 (16.8%)	15 (16.7%)	3 (17.6%)	>0.99	-
Gout history	7 (6.5%)	7 (7.8%)	0 (0.0%)	0.59	-
Urinary tract infection	8 (7.5%)	7 (7.8%)	1 (5.9%)	>0.99	-
Pneumonia	27 (25.2%)	21 (23.3%)	6 (35.3%)	0.36	-
Chronic heart failure	24 (22.4%)	18 (20.0%)	6 (35.3%)	0.21	-
Chronic kidney disease	13 (12.1%)	10 (11.1%)	3 (17.6%)	0.43	-
Joint characteristics					
Number of joints affected	1.0 (1.0-2.0)	1.0 (1.0-2.0)	1.0 (1.0-1.0)	0.41	0.82
Knee	92 (86.0%)	79 (87.8%)	13 (76.5%)	0.25	-
Bilateral knee	19 (17.8%)	18 (20.0%)	1 (5.9%)	0.3	-
Maximum body temperature, °C	37.6 (37.1-38.2)	37.6 (37.2-38.2)	37.5 (37.1-38.2)	0.98	0.02
Blood tests at the time of puncture					
White blood cell, ×1000/µL	8.4 (7.2-10.5)	8.4 (7.2-10.5)	8.3 (7.7-10.9)	0.57	-0.56
CRP, mg/dL	10.9 (5.1-17.5)	10.9 (4.4-17.5)	10.8 (7.1-14.9)	0.59	-0.54
Calcium, mg/dL	8.5 (8.2-9.0)	8.6 (8.2-9.0)	8.4 (8.2-8.8)	0.66	0.44
Albumin, g/dL	2.9 (2.4-3.3)	2.9 (2.4-3.3)	2.6 (2.3-3.4)	0.6	0.53
Uric acid, mg/dL	4.7 (3.8-6.6)	4.5 (3.7-6.6)	6.1 (5.1-6.8)	0.048	-1.98
X-ray findings					
Calcification	57 (61%)	49 (62%)	8 (53%)	0.57	-
Osteoarthritis	58 (60%)	47 (58%)	11 (73%)	0.39	-

Table 2 shows treatments and outcomes. No patients received intra-articular injections. The most common treatment was oral nonsteroidal anti-inflammatory drugs, followed by oral acetaminophen. The median time from symptom onset to clinical improvement was 4.0 days in both groups. There were no statistically significant differences between the two groups in terms of treatments or outcomes.

**Table 2:** Treatment and outcomes Data are shown as numbers (%) and median (IQR) unless otherwise specified. P-values were calculated using the Mann-Whitney U test for continuous variables and Fisher's exact test for categorical variables. A two-sided P-value of less than 0.05 was considered statistically significant. The test statistic shown is the z-value from the Mann-Whitney U test; Fisher's exact test does not yield a test statistic (-). NSAIDs, non-steroidal anti-inflammatory drugs; CRP, C-reactive protein; IQR, interquartile range

Variables	Total (n=107)	Pseudogout-only group (n=90)	Gout-superimposed group (n=17)	P-value	Test statistic
Treatments, n (%)					
Intra-articular injections	0 (0.0%)	0 (0.0%)	0 (0.0%)	-	-
Oral medications					
NSAIDs	73 (68.2%)	62 (68.9%)	11 (64.7%)	0.78	-
Acetaminophen	41 (38.3%)	31 (34.4%)	10 (58.8%)	0.1	-
Glucocorticoid	5 (4.7%)	5 (5.6%)	0 (0.0%)	>0.99	-
Colchicine	3 (2.8%)	2 (2.2%)	1 (5.9%)	0.41	-
Anti-uric acid drug	1 (0.9%)	1 (1.1%)	0 (0.0%)	>0.99	-
CRP after 3 days, mg/dL	9.0 (4.8-13.2)	9.1 (3.9-14.8)	7.8 (4.8-11.0)	0.56	0.58
CRP after 5 days, mg/dL	3.5 (1.2-7.0)	3.4 (1.2-7.0)	4.5 (2.0-15.0)	0.22	-1.22
CRP after 7 days, mg/dL	2.5 (0.6-5.9)	2.3 (0.6-5.6)	2.8 (2.2-12.6)	0.41	-0.82
Duration of symptom, days	4.0 (3.0-6.0)	4.0 (3.0-6.0)	4.0 (2.0-5.0)	0.15	1.44

## Discussion

In the present study, we evaluated synovial fluid crystals in patients diagnosed with pseudogout and identified concomitant MSU crystals in approximately 16% of patients, a higher proportion than previously reported. Comparison between the pseudogout-only and gout-superimposed groups revealed no statistically significant differences in patient backgrounds, treatments, or outcomes, except for higher serum uric acid levels in the gout-superimposed group. Among patients with concomitant gout and pseudogout, the condition was more common in elderly women and predominantly affected the knees.

Polarized light microscopy revealed the coexistence of CPPD and MSU crystals within the same specimens; the two crystal types were rarely seen within a single microscopic field and were identified only through careful examination of multiple fields. Prior histopathological studies of crystal-associated arthropathy have described marked inflammatory cell infiltration with features of chronic synovitis, fibrosis, and cartilage degeneration [2]. Both CPPD and MSU crystals have also been reported to activate shared inflammatory pathways, including nucleotide-binding oligomerization domain-like receptor family, pyrin domain-containing 3 (NLRP3) inflammasome-mediated interleukin-1β production, which may amplify and sustain synovial inflammation [2]. Differences in crystal deposition patterns within cartilage and fibrous tissue have been suggested to contribute to chronicity and promote osteoarthritis progression [2]. Taken together with these earlier observations, the coexistence of CPPD and MSU crystals is unlikely to be incidental and may instead be related to shared factors such as aging, joint degeneration, and metabolic abnormalities [2,11]. However, the present study was limited to polarized light microscopy of synovial fluid and did not include tissue biopsy, histopathologic evaluation, or measurement of inflammatory mediators. Accordingly, the histopathologic and mechanistic interpretations discussed above are based on prior literature rather than directly demonstrated by our own data and should therefore be interpreted cautiously.

Previous investigations in patients with coexistence of pseudogout and gout have focused on patients with gout, in whom CPPD deposition was identified incidentally during synovial fluid analysis [8,11-13]. In these studies, the reported coexistence rates were derived predominantly from gout cohorts and are not directly comparable with the prevalence observed in our cohort because the source populations and denominators differed. Even in a prospective cohort study of 1,544 patients with gout, concomitant CPPD was observed in 2.4% of patients at the time of initial gout diagnosis, and CPPD was detected in 5.8% during five years of follow-up after gout diagnosis [8]. Similarly, a retrospective analysis of 6,983 synovial fluid samples containing MSU crystals from patients with various conditions involving fluid accumulation identified mixed CPPD crystal arthropathy in 171 patients (2.5%) [7]. In addition, a recent histopathological analysis of gouty tophi identified concurrent CPPD crystals in nine of 156 patients with gout (5.7%) [11]. These results indicate that the coexistence of CPPD and MSU crystals has generally been considered uncommon when assessed from the perspective of gout. In contrast, data evaluating the presence of MSU crystals in patients initially diagnosed with pseudogout are limited. To our knowledge, no studies have systematically evaluated MSU crystal deposition in cohorts primarily diagnosed with pseudogout using polarized light microscopy. Although several case reports have described the coexistence of pseudogout and gout, these reports cannot establish prevalence or clinical relevance [9,13].

The higher coexistence rate observed in our pseudogout cohort suggests that concomitant pseudogout and gout may be underrecognized in patients presenting with pseudogout. Both groups in the present study consisted mainly of elderly women, and the knee was the most frequently affected joint. In previous studies focusing on patients with gout, coexistence of pseudogout and gout has often been reported in the upper limbs. In routine clinical practice, acute monoarthritis of the upper limbs or toes can be diagnosed as gout, and joint aspiration with crystal identification using polarized light microscopy would not routinely be performed. When clinicians encounter acute monoarthritis of the knee, joint aspiration is often performed because symptomatic improvement may be expected with aspiration alone. Additionally, joint aspiration may be needed to exclude bacterial arthritis in patients presenting with fever. Because our study population primarily comprised patients with suspected pseudogout, who are typically older and more often female, the observed coexistence rate was likely higher than that reported in previous studies. In such populations, a certain proportion of patients are expected to have hyperuricemia. Serum uric acid levels were significantly higher in patients with concomitant gout than in those with pseudogout alone (P=0.048).

In clinical practice, joint aspiration in suspected pseudogout is commonly undertaken to evaluate fever or marked inflammation. Management of pseudogout is typically short-term and symptom-directed, whereas gout warrants long-term urate-lowering therapy to reduce the risk of recurrent flares. Accordingly, detection of concomitant MSU crystals may facilitate recognition of possible coexisting gout. However, the present study did not determine whether this finding should prompt urate-lowering therapy or whether it affects subsequent flare risk or other clinical outcomes. These findings support careful assessment for both CPPD and MSU crystals during synovial fluid microscopy, and prospective studies are needed to determine the clinical consequences of detecting concomitant crystals.

This study has several strengths. First, it included consecutive patients with compatible clinical presentations and crystal-proven pseudogout. Second, all included synovial fluid specimens were assessed for both CPPD and MSU crystals, allowing concomitant crystal deposition to be evaluated systematically rather than incidentally. Third, the study was conducted in a real-world tertiary emergency care setting and therefore reflects routine clinical practice. Finally, the cohort was relatively large compared with the predominantly case-based literature evaluating concomitant MSU crystals in patients with pseudogout.

Several limitations of this study should be acknowledged. First, this was a single-center retrospective study, which may limit the external validity and generalizability of our findings to other clinical settings and populations. Second, the indication for synovial fluid aspiration was not standardized. In routine clinical practice, joint aspiration is not always performed in patients with clinically typical gout attacks, such as acute inflammation of the first metatarsophalangeal joint. As a result, patients undergoing joint aspiration may represent a selected population, introducing potential selection and referral bias. Third, crystal identification was performed by multiple clinical laboratory technologists on a rotating basis. Although polarized light microscopy is the diagnostic standard for crystal-induced arthritis, interobserver variability related to differences in technical expertise cannot be fully excluded and may have affected crystal detection. Formal interobserver reliability was not assessed. Moreover, because of the retrospective design, information on the number and training of individual examiners and the interval from aspiration to microscopic examination was unavailable. Fourth, because of the retrospective design, CRP values on prespecified days were incomplete and were partially supplemented using nearby clinical values; they were ultimately available for 54, 48, and 56 patients on days 3, 5, and 7, respectively. Therefore, the findings regarding CRP trajectories should be interpreted as exploratory. Data were also incomplete for several variables in Table 1: serum calcium (45.8% missing), uric acid (21.5% missing), albumin (16.8% missing), and radiographic findings (10.3-12.1% missing). These variables were analyzed on an available-case basis. Fifth, comprehensive data on background factors such as body mass index, alcohol consumption, smoking status, and diuretic use were not available in this retrospective single-center setting, which limits further evaluation of potential confounders. Sixth, the small number of patients with concomitant MSU crystals may have limited the statistical power to detect between-group differences. Seventh, long-term clinical outcomes could not be systematically evaluated. Although the detection of concomitant MSU crystals in patients with CPPD arthritis may have facilitated appropriate therapeutic intervention, we could not assess recurrent gout or pseudogout attacks, subsequent initiation of urate-lowering therapy, or whether crystal identification affected long-term clinical outcomes. Future multicenter prospective studies with standardized criteria for joint aspiration, standardized crystal assessment, and longitudinal follow-up are warranted to more accurately determine the true prevalence, clinical significance, and prognostic impact of crystal coexistence. Several additional considerations warrant emphasis. Because the gout-superimposed group comprised only 17 patients, the absence of statistically significant between-group differences should not be interpreted as evidence of equivalence and may reflect a type II error. Multivariable modeling was not performed because estimates based on so few patients would have been unstable and prone to overfitting. Although the advanced age and female predominance of the cohort (median age, 86 years; 74% women) are consistent with the epidemiologic profile of CPPD disease, they also reflect the referral population of this tertiary center and may limit the generalizability of the 15.9% prevalence estimate to other settings. Because crystal identification was complete for all 107 patients, missing laboratory and radiographic data did not affect the primary prevalence estimate. By contrast, the observed between-group difference in serum uric acid was based on an unadjusted available-case analysis, reached only nominal statistical significance (P=0.048), and should be considered exploratory. Because no adjustment was made for multiple comparisons, this isolated finding should be regarded as hypothesis-generating rather than confirmatory. Finally, crystal abundance was not assessed using a standardized quantitative method; the apparent predominance of CPPD over MSU crystals reflects a qualitative microscopic impression.

## Conclusions

This study demonstrated that concomitant MSU crystals were identified in approximately 16% of patients with crystal-proven pseudogout who underwent synovial fluid analysis. This proportion was higher than the rates reported in previous studies, which predominantly evaluated CPPD crystals in gout cohorts. These findings suggest that coexisting gout may be underrecognized among patients presenting with pseudogout. Notably, all cases of concomitant gout identified in our cohort represented the first clinically recognized episode of gout. Systematic assessment for MSU crystals during synovial fluid microscopy may facilitate recognition of concomitant gout in patients with pseudogout; however, prospective studies are needed to determine whether such identification changes clinical management or improves patient outcomes.

## References

[1] McCarthy GM, Dunne A (2018). Calcium crystal deposition diseases - beyond gout. Nat Rev Rheumatol.

[2] Pascart T, Filippou G, Lioté F, Sirotti S, Jauffret C, Abhishek A (2024). Calcium pyrophosphate deposition disease. Lancet Rheumatol.

[3] He Q, Mok TN, Sin TH (2023). Global, regional, and national prevalence of gout from 1990 to 2019: age-period-cohort analysis with future burden prediction. JMIR Public Health Surveill.

[4] Ooyama H, Ooyama K, Moromizato H, Fujimori S (2020). Recent trend in onset age of gout in Japan. Japan Med Assoc J.

[5] Qi H, Chen H, Dalbeth N (2026). Two-decade trajectories of gout: a multicentre cohort study of 10 658 Chinese patients with gout. Rheumatology (Oxford).

[6] Li Y, Piranavan P, Sundaresan D, Yood R (2019). Clinical characteristics of early-onset gout in outpatient setting. ACR Open Rheumatol.

[7] Heselden EL, Freemont AJ (2016). Synovial fluid findings and demographic analysis of patients with coexistent intra-articular monosodium urate and calcium pyrophosphate crystals. J Clin Rheumatol.

[8] Perez-Ruiz F, Modesto-Caballero MDC, Herrero-Beites AM (2024). Prevalence and factors associated to calcium pyrophosphate arthritis in patients with gout. Explor Musculoskeletal Dis.

[9] Yoo Y, Seo YJ, Huh M, Yoo JH, Yun KH, Kim SJ (2011). Gout and coexisting pseudogout in the knee joint. Knee Surg Sports Traumatol Arthrosc.

[10] Jaccard YB, Gerster JC, Calame L (1996). Mixed monosodium urate and calcium pyrophosphate crystal-induced arthropathy. A review of seventeen cases. Rev Rhum Engl Ed.

[11] Gjeorgjievski SG, Dermawan JK, Reith JD, Kilpatrick SE (2025). Combined gout and pseudogout demonstrate unique clinicopathologic features compared to those with only gout. Am J Surg Pathol.

[12] Mohr W, Görz E (2000). Mixed tophi. Calcium pyrophosphate dihydrate crystals in gout tophi (German). Z Rheumatol.

[13] Ea HK, Gauffenic A, Nguyen QD (2021). Calcium pyrophosphate dihydrate crystal deposition in gouty tophi. Arthritis Rheumatol.

